# DPA promotes hBMSCs osteogenic differentiation by miR-9-5p/ERK/ALP signaling pathway

**DOI:** 10.7150/ijms.77729

**Published:** 2022-10-24

**Authors:** Xiang Gao, Guanhao Hong, Weiqiang Zhan, Tianfeng Liu, Shouquan Yan, Mingzhu Deng, Chenlin Tu, Peng Li

**Affiliations:** 1 Stem Cell Research and Cellular Therapy Center, Affiliated Hospital of Guangdong Medical University, Zhanjiang 524001, China.; 2 Orthopedic Center, Affiliated Hospital of Guangdong Medical University, Zhanjiang, 524001, China.; 3 Scientific Research Department, Affiliated Hospital of Guangdong Medical University, Zhanjiang, 524001, China.

**Keywords:** DPA, osteogenic differentiation, hBMSCs, miR-9-5p, ERK, ALP

## Abstract

Docosahexaenoic acid (DHA) has been reported potentiate osteogenic differentiation, while Docosapentaenoic acid (DPA), another Omega-3 fatty acid, its contribution to the osteogenic differentiation of human bone-marrow-derived mesenchymal stromal cells (hBMSCs) is not entirely elucidated. The Alizarin Red S (ARS) staining and the expression of osteogenesis‑associated genes were analyzed during osteogenic induction by DPA. Then, bioinformatics analysis and dual luciferase reporter assays were investigated to confirm the interactions between miR-9-5p and alkaline phosphatase (ALP). miR-9-5p mimics / inhibitor were transfected to human hBMSCs and the osteogenic assay above was also performed. Furthermore, DPA significantly promoted the phosphorylation of ERK via miR-9-5p. PD98059, a highly specific and potent ERK1/2 inhibitor, inhibited the activation of ALP and partially reversed the role of DPA during osteogenic differentiation. These data indicated that DPA promoted osteogenic differentiation of hBMSCs potentially through miR-9-5p/ERK/ALP signaling pathway, providing a potentially useful therapeutic strategy for patients to improve bone loss.

## Introduction

Osteoporosis is the most common age-related bone disease, characterized by a decrease of bone mass and deterioration of bone architecture, leading to osteoporosis and fracture risk in older people 1. Almost 200 million patients suffer from osteoporosis and approximately 8.9 million concurrent symptom caused by osteoporotic fracture with greatly increased risk of death making osteoporosis to be a worldwide problem 2. Osteoporosis is mainly caused by the imbalance between bone formation and bone resorption during bone metabolism, and bone marrow mesenchymal stem cells (BMSCs) play an important role in bone repair 3. The regulatory mechanisms of BMSCs differentiation into osteoblasts provide new strategies for the prevention and treatment of osteoporosis.

miRNAs are small single-stranded non-coding RNA molecules containing 21 to 25 nucleotides and responsible for regulating gene expression 4. Researchers have found that miRNAs promote osteogenic differentiation and mineralization by stimulating the expression of relevant mRNA and protein 5. Recently, B Arumugam et al. demonstrated that the expression of miR-21 in mouse mesenchymal stem cells was increased after treated with syringic acid, and the inhibition of Smad7 expression increased the expression of Runx2, leading to increased osteoblast differentiation 6. Yin et al. found that miR-135-5p promoted osteogenic differentiation and mineralization of MC3T3-E1 cells by blocking the translation of hypoxia-induction-factor 1α (HIF-1α) inhibitor and the calcification of ALP activity and the levels of runt‐related transcription factor2 (Runx2), osteopontin (OPN) and osteocalcin (OCN) were also increased 7. However, the role of miRNAs in osteoblastic differentiation of BMSCs still needs further investigation.

DPA, a lesser known member of the omega-3 family, is another type of long chain omega-3 fatty acid, which is structurally similar to eicosapentaenoic acid (EPA) with the same number of double bonds, but two more carbon chain units 8. Like EPA and DHA, DPA is found in fish, seafood, and certain omega-3 supplements and to a lesser extent in meats and poultry 9. DPA omega 3's biological properties have not been studied to the same extend as EPA and DHA which have stolen the spotlight when it comes to health promotion 10. DPA is involved in altering the gene expression especially genes that reduce the synthesis of fat and also play a part in reducing the expression of inflammatory genes 11. Previous study has demonstrated that DHA promotes osteogenic differentiation of MSCs via increasing Akt activity 12. While as a member of ω-3 polyunsaturated fatty acids like DHA, the biological role of DPA in osteoporosis remains unclear.

Here in our study, we aimed to determine whether DPA promotes hBMSCs osteogenic differentiation and to reveal the underlying molecular mechanisms. We found that DPA promotes hBMSCs osteogenic differentiation by miR-9-5p/ERK/ALP signaling pathway, which providing a new therapeutic target for the treatment of osteoporosis.

## Methods

### Isolation and culture of primary hBMSCs

Primary hBMSCs were isolated from patients undergoing hip replacements as previous described 13. hBMSCs were isolated from bone marrow and separated by density gradient centrifugation in Ficoll-Histopaque (d = 1.077 g/ml; Pharmacia, Uppsala, Sweden) and then cultured in low-glucose Dulbecco's MEM medium with 10% FBS and 1% Penicillin/Streptomycin and non-adherent cells were removed after 72 hr. This study was approved by the ethics committee of the Medical Ethical Committee of the Affiliated Hospital of Guangdong Medical University.

### Induction of osteogenic differentiation

For osteogenic differentiation, hBMSCs were cultured with osteogenic induction medium (OIM) including 10 mM β-glycerophosphate, 100 nM dexamethasone and 50 µM ascorbate-2-phosphate for 14 days and the differentiation capacity was evaluated by ARS staining or ALP staining following the manufacturer's instructions.

### hBMSC transfection

miR-9-5p mimics, inhibitor and negative control were purchased from GeneChem. The sequences were as follows: miR-9-5p mimic NC: 5′- UUCUCCGAACGUGUCACGUTT-3′; miR-9-5p mimic: 5′-TCTTTGGTTATCTAGCTGTATGAAA-3′; miR-9-5p inhibitor NC: 5′-CAGUACUUUUGUGUAGUACAA-3′; and miR-9-5p inhibitor: 5′- UCAUACAGCUAGAUAACCAAAGA-3′. hBMSCs were transfected with miR-9-5p mimic, inhibitor and negative control with Lipofectamine® 2000 under osteogenic induction medium for 14 days at a final concentration of 100 nM.

### Quantitative real-time PCR (qRT-PCR)

Total RNA was extracted with Trizol reagent and miRNA was extracted by a miRNA purification kit and was reverse transcript into cDNA according the manufacturer's instructions. Real-time quantitative PCR was performed using the SYBR Green reagent. The primers are shown in the list:

- RUNX2-Forward: 5'-CTCTACTATGGCACTTCGTCAGG-3';
- RUNX2-Reverse: 5'-TCAGCGTCAACACCATCATTC-3';
- OPN-Forward: 5'-GGGAAGGACAGTTATGAAACGAG-3';
- OPN-Reverse: 5'-CCTGACTATCAATCACATCGGAAT-3';
- OCN-Forward: 5'-GAGGGCAGCGAGGTAGTGAA-3';
- OCN-Reverse: 5'-TAGACCGGGCCGTAGAAGC-3';
- ALP-Forward: 5'-CTTCAAACCGAGATACAAGCACTC-3';
- ALP-Reverse: 5'-CGTTGTTCCTGTTCAGCTCGTAC-3';
- GAPDH-Forward: 5'-GTCTCCTCTGACTTCAACAGCG-3';
- GAPDH-Reverse: 5'-ACCACCCTGTTGCTGTAGCCAA-3'.

### Western blot

Total protein was extracted from hBMSCs using RIPA buffer containing protease inhibitors and phosphatase inhibitors. Then the same amount of protein was separated by a 10%-15% SDS-PAGE electrophoresis, and then transferred to a polyvinylidene fluoride (PVDF) membrane with a 5% skim milk blocking buffer for 1 hr at room temperature. Next, the membranes were incubated with primary antibodies at 4 °C overnight and then incubated with secondary antibodies for 1 h at room temperature and the blots were detected using Pierce ECL Western Blotting Substrate.

### Bioinformatic analysis of miRNA sequencing

RNA Isolation Total RNA was isolated according to the protocol. The concentration and integrity of the extracted total RNA was estimated by Qubit 3.0 Fluorometer (Invitrogen, Carlsbad, California), and Agilent 2100 Bioanalyzer (Applied Biosystems, Carlsbad, CA), respectively. Library preparation for small RNA Sequencing was prepared with approximately 100 ng of total RNA using VAHTSTM Small RNA Library Prep Kit for Illumina® (Vazyme Biotech). Briefly, RL3 Adaptor was directly and specifically ligated to 3' end of miRNA, siRNA and piRNA. Next, the RT primer was hybridized to the excess of RL3 Adaptor after 3' ligation reaction to transform the single-stranded DNA adaptor into a double-stranded DNA molecule. Then RL5 Adaptor was ligated to 5' end of miRNA, siRNA and piRNA. Subsequently, the first strand cDNA was synthesized using M-MuLV Reverse Transcriptase (RNase H free) and PCR amplification was performed using Universal Primer for illumina and Index (X) Primer as barcode. Finally, the PCR product was purified and the target DNA fragments (140~160bp) were chosen using polyacrylamide gel or Ampure XP beads. The library quality and concentration were assessed by utilizing a DNA 1000 chip on an Agilent 2100 Bioanalyzer. Accurate quantification for sequencing applications was determined using the qPCR-based KAPA Biosystems Library Quantification kit (Kapa Biosystems, Inc., Woburn, MA). Each library was diluted to a final concentration of 2 nM and pooled equimolar prior to clustering. 50 bp single-end (SE) sequencing was performed on all samples. For miRNA expression analysis, exceRpt was used to estimate the miRNA expression in miRBase. Alternatively, novel miRNAs were identified with miRDeep2. TMM (trimmed mean of M-values) was used to normalize the gene expression. Differentially expressed genes were identified using the edgeR program. Genes showing altered expression with p < 0.05 and more than 1.5-fold changes were considered differentially expressed.

### Dual-luciferase reporter assay

The potential binding between miR-9-5p and ALP was predicted with TargetScan (http://www.targetscan.org) and was confirmed by dual-luciferase reporter assay. The wild-type and the mutant sequences of ALP 3′-UTR were inserted into a luciferase reporter plasmid. 293T cells were seeded and co-transfected with miR-NC or miR-9-5p mimics using Lipofectamine 2000 (Invitrogen) and the luciferase activities were further determined with a Luciferase Reporter System.

### Statistical analysis

All data were presented as the mean ± standard deviation by GraphPad Prism software and a t test was used to compare the differences between two groups. p < 0.05 was considered to be statistically significant.

## Results

### DPA promoted osteoblast differentiation of hBMSCs

**Figure 1:**
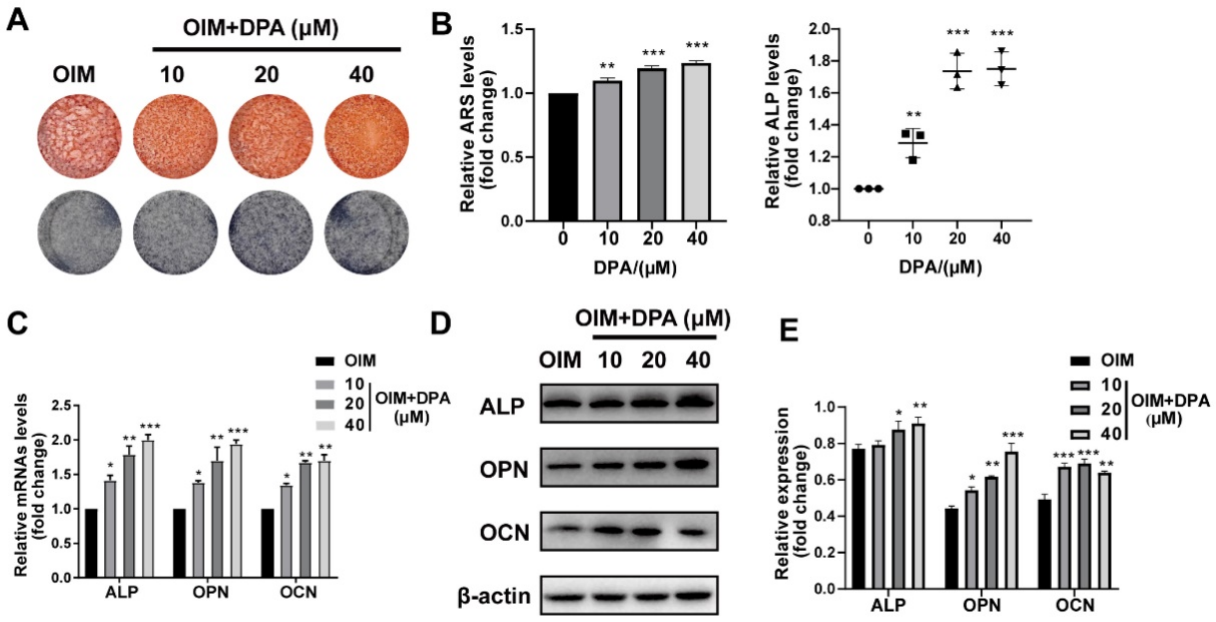
** DPA promoted osteogenic differentiation of hBMSCs. (A-B)** Under osteogenic inducing medium (OIM) conditions, hBMSCs were treated with the indicated concentrations of DPA for 14 days and the mineralization was assessed by Alizarin Red S staining (ARS) and ALP activity. **(C-E)** The osteogenesis-related gene expression (including ALP, OPN and OCN) of DPA-treated (0, 10, 20, 40 µM) hBMSCs was measured by qRT-PCR (C) and western blot (D-E) assays. *p < 0.05, **p < 0.01, ***p < 0.001 vs OIM group.

To investigate the effect of DPA on the osteogenesis, hBMSCs were treated with varying concentrations of DPA (0, 10, 20, and 40 μM) and stained with Alizarin Red S and ALP staining. After 14 days of osteogenic induction by OIM medium, the production of mineralized nodules and ALP staining were also greatly higher in the DPA treating group with a dose-dependent manner (Figure 1A-B). To further confirm the osteogenic induction, we investigated how DPA affects osteogenic-related gene expression (including ALP, OPN and OCN) by qPCR and Western blotting. The data showed that the expression of ALP, OPN, and OCN was definitely increased in the DPA group (Figure 1C-E). These data above indicated that DPA promotes osteogenic differentiation in hBMSCs.

### miR-9-5p inhibited osteogenic differentiation of hBMSCs

**Figure 2:**
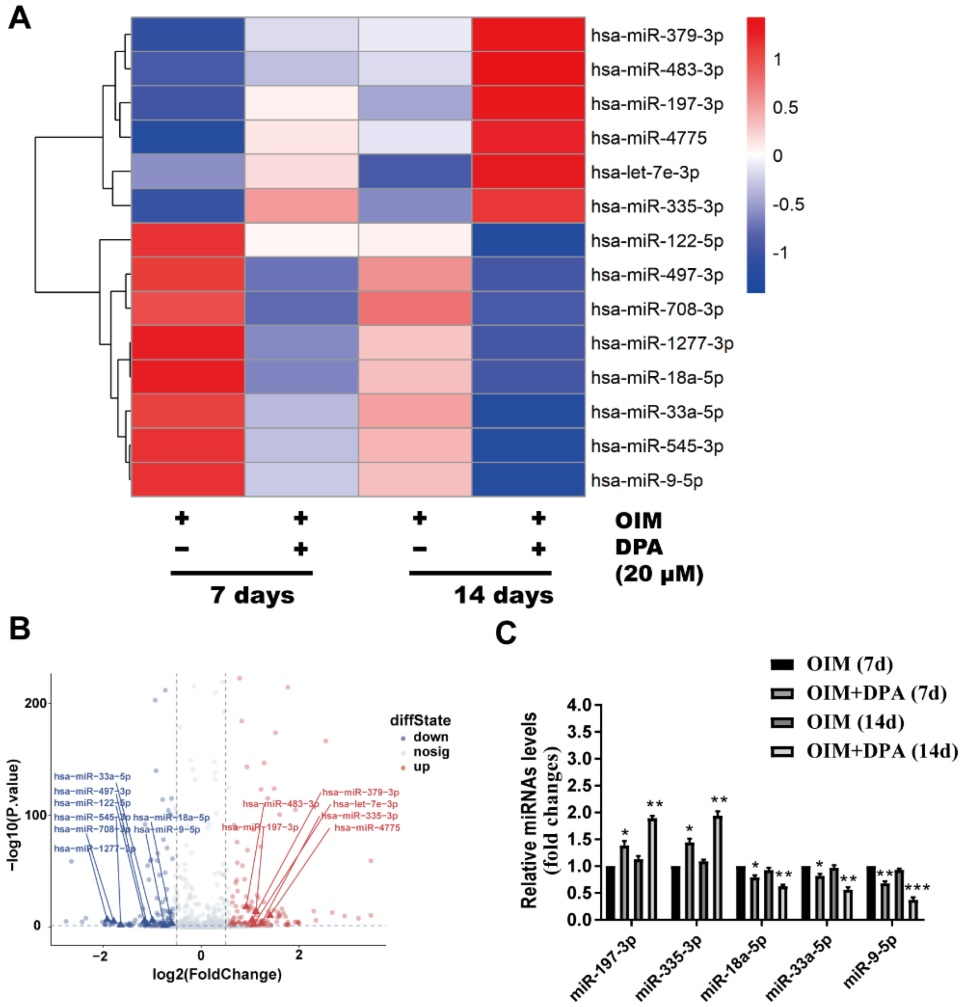
** DPA decreased the expression of miR-9-5p in hBMSCs.** Heatmap analysis **(A)** and volcano plot **(B)** of differentially expressed miRNAs after treatment for 7 and 14 days with DPA (20 µM) induced by OIM medium in hBMSCs. **(C)** qRT-PCR validation of miRNAs expression in the hBMSCs with or without DPA treatment. *p < 0.05, **p < 0.01, ***p < 0.001 vs OIM group.

To further explore the molecular mechanism of DPA in osteogenic differentiation, hBMSCs cultured in OIM medium for 7 and 14 days with and without DPA (20 μM) induction were screened by miRNA sequencing analysis for dysregulated miRNAs. Among these dysregulated miRNAs, miR-9-5p expression was significantly decreased in hBMSCs during DPA treatment and was further validated by qPCR analysis (Figure 2A-C).

**Figure 3:**
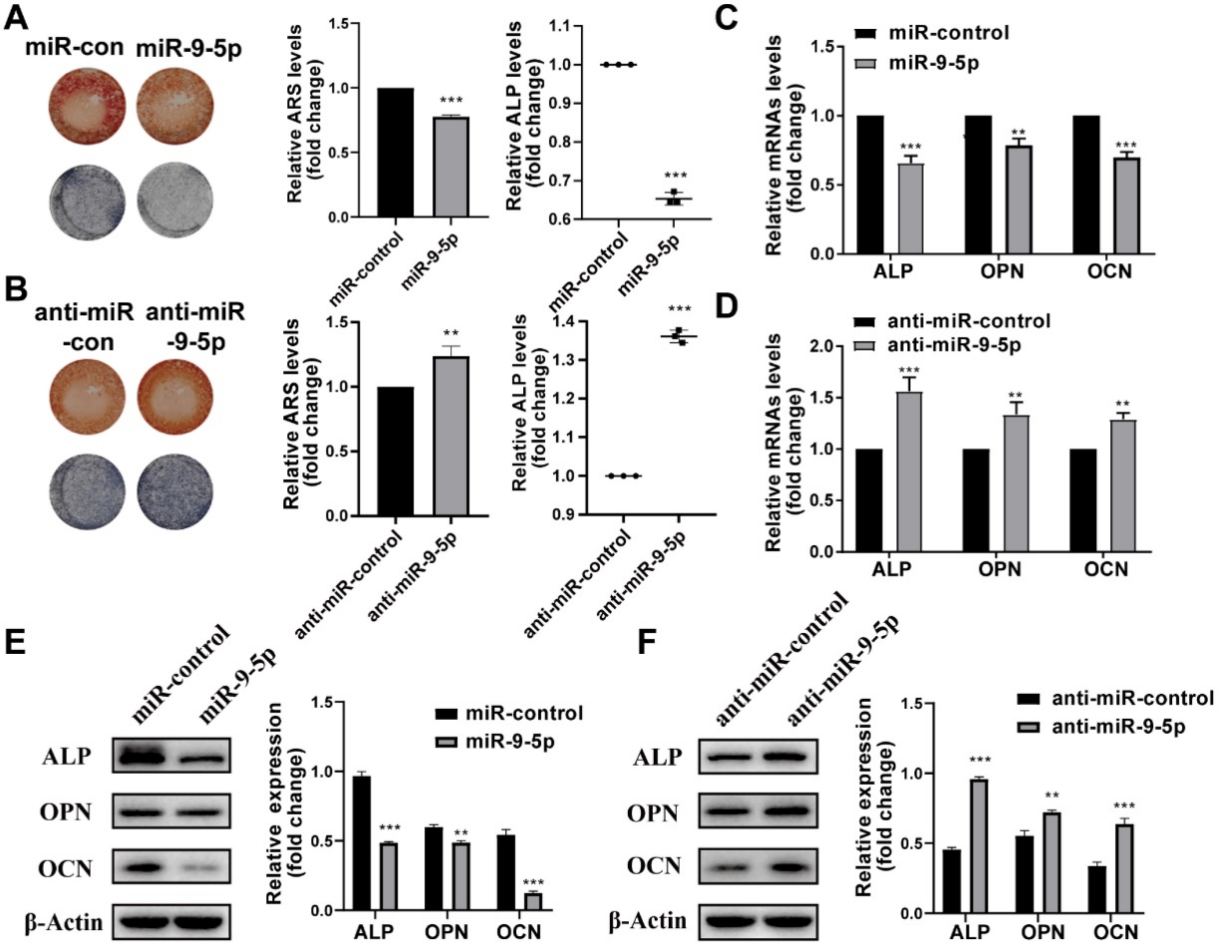
** miR-9-5p inhibited osteogenic differentiation of hBMSCs. (A-B)** The mineralization of hBMSCs transfected with miR-9-5p mimic and miR-9-5p inhibitor (anti-miR-9-5p) was assessed by Alizarin Red S staining and ALP activity. **(C-F)** The osteogenesis-related gene expression (including ALP, OPN and OCN) of hBMSCs transfected with miR-9-5p mimic and miR-9-5p inhibitor (anti-miR-9-5p) was measured by qRT-PCR (C-D) and western blot (E-F) assays. **p < 0.01, ***p < 0.001 vs miR-control.

Then the effect of miR-9-5p on hBMSCs during osteogenic differentiation was investigated. We found that the transfection of miR-9-5p mimic and miR-9-5p inhibitor (anti-miR-9-5p) could separately decrease and increase the mineralization of hBMSCs by ARS and ALP staining, respectively (Figure 3A-B). Moreover, the results of qPCR and Western blotting also showed that the expression of ALP, OPN, and OCN was significantly reduced in hBMSCs transfected with miR-9-5p mimic while increase with miR-9-5p inhibitor, respectively (Figure 3C-F). Taken together, our results suggested that miR-9-5p played a negative regulatory role in osteoblast differentiation and subsequently suppressed mineralization.

### DPA promotes osteogenic differentiation through the miR-9-5p/ERK1/2 pathway

**Figure 4:**
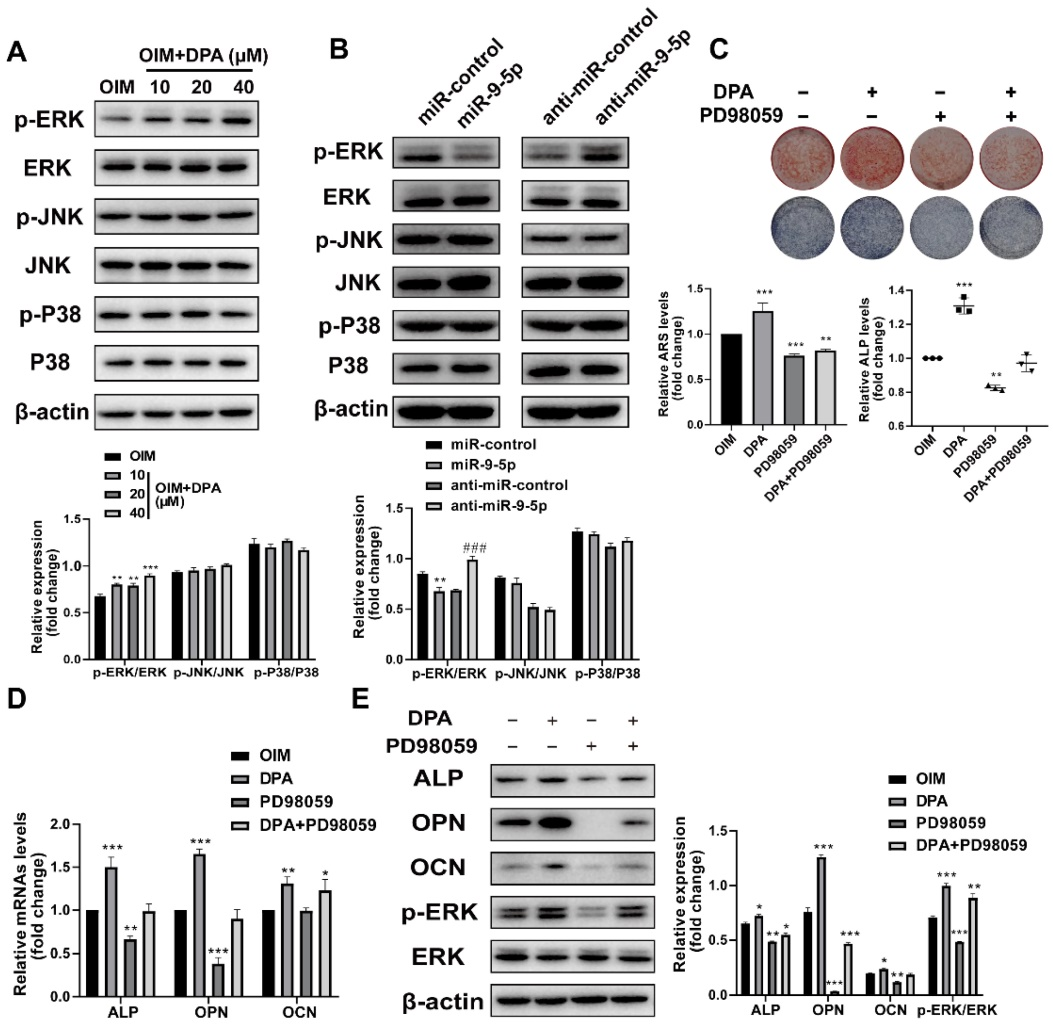
** DPA promoted osteogenic differentiation via ERK signaling pathway. (A)** The expression of phosphorylated-ERK, JNK and p38 of hBMSCs treated with DPA (0, 10, 20, 40 µM). **(B)** The expression of phosphorylated-ERK, JNK and p38 of hBMSCs treated with miR-9-5p mimic and miR-9-5p inhibitor (anti-miR-9-5p). **(C)** The mineralization of hBMSCs pretreated with ERK specific inhibitor PD98059 for 24 hr with or without DPA for 7 days was assessed by Alizarin Red S staining and ALP activity. **(D-E)** The osteogenesis-related gene expression (including ALP, OPN and OCN) of hBMSCs pretreated with ERK specific inhibitor PD98059 for 24 hr with or without DPA for 7 days was measured by qRT-PCR (D) and western blot (E) assays. *p < 0.05, **p < 0.01, ***p < 0.001 vs OIM group.

Previous studies have demonstrated that the ERK/MAP kinase pathway play an important role in bone and stimulates osteoblastogenesis 14, we then investigated whether MAPK activation mediates DPA-induced osteogenic differentiation. Interestingly, the expression of p-ERK1/2 increased whilst the levels of p-p38 and p-JNK showed no changes with DPA for 48 h (Figure 4A). Moreover, the expression ERK1/2 phosphorylation was suppressed and promoted when miR-9-5p was overexpressed or inhibited, respectively (Figure 4B).

Then the effects of the specific ERK1/2 inhibitor treatment of PD98059 (10 μM) on DPA induced osteoblast differentiation were investigated as well. The hBMSCs were pretreated with PD98059 for 24 h and the medium was replaced with DPA, next, the mineralization was analyzed by ARS and ALP staining and the expression of osteoblast-related proteins was detected by qPCR and western blotting. The results showed that decreased mineral deposition in PD98059 inhibitor treated cells compared with negative control (Figure 4C) and the upregulation of ALP and OPN were significantly prevented by treatment with PD98059 (Figure 4D-E). These data suggested that the DPA induced osteoblast differentiation from hBMSCs was mediated by ERK1/2 signaling.

**Figure 5:**
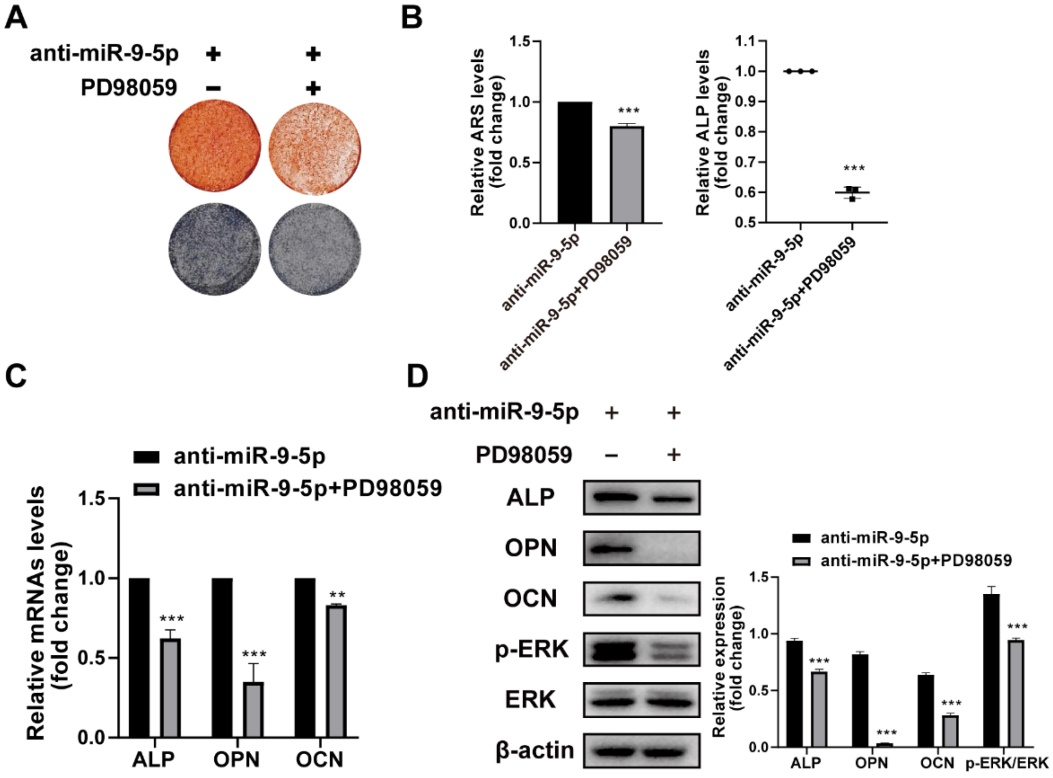
** miR-9-5p promoted osteogenic differentiation through the ERK signaling pathway. (A-B)** The mineralization of hBMSCs pretreated with or without ERK specific inhibitor PD98059 for 24 hr with miR-9-5p inhibitor was assessed by Alizarin Red S staining and ALP activity. **(C-D)** The osteogenesis-related gene expression (including ALP, OPN and OCN) of hBMSCs pretreated with or without ERK specific inhibitor PD98059 for 24 hr with miR-9-5p inhibitor was measured by qRT-PCR (C) and western blot (D) assays. *p < 0.05, **p < 0.01, ***p < 0.001 vs anti-miR-9-5p group.

To further demonstrate the DPA promotes osteogenic differentiation through the miR-9-5p/ERK1/2 pathway, the hBMSCs were with PD98059 for 24 h and the effect of miR-9-5p inhibitor on hBMSCs was investigated. The mineralization was assessed by ARS staining and ALP activity and the expression of osteoblast-related markers was evaluated by qPCR and western blotting. The ARS and ALP staining showed that the mineralization was suppressed under PD98059 conditions (Figure 5A-B). Similarly, the mRNA and protein expression of ALP, OPN and OCN were decreased by PD98059 treatment (Figure 5C-D). Apparently, DPA could affect osteoblasts development through interference in miR-9-5p/ERK1/2 pathway.

### ALP is a direct target of miR-9-5p

**Figure 6:**
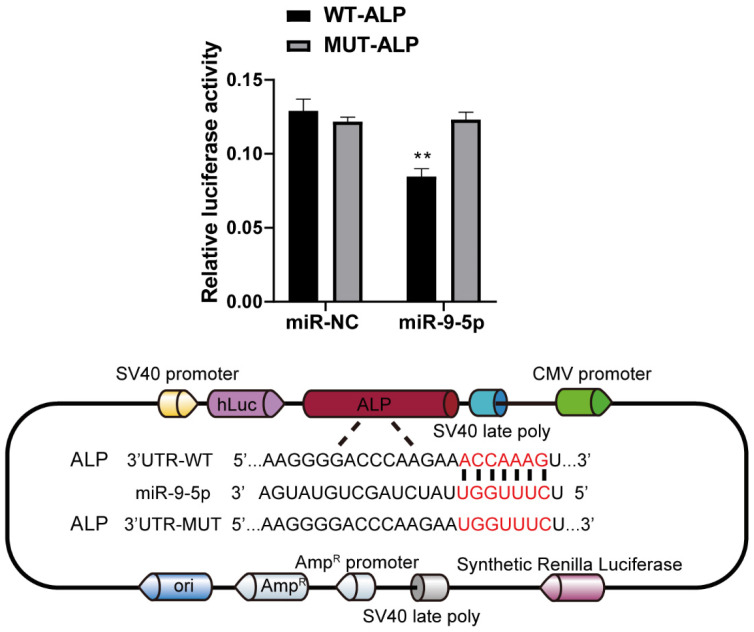
** ALP is a direct target of miR-9-5p.** Left 293T cells were transfected with miR-9-5p, or mimic control and then with the indicated ALP 3′UTR reporter or an empty vector and a Renilla luciferase control vector. The firefly luciferase activity was measured after 48 h and was normalized to Renilla luciferase activity and to the empty vector. Right showed the potential binding site of ALP 3′UTR reporter and miR-9-5p. **p < 0.01 vs WT-ALP group.

To decipher the downstream genes regulated by miR-9-5p, the possible target was predicted by with the help of TargetScan (http://www.targetscan.org) and was confirmed by dual-luciferase reporter assay. As a result of the analyses, we selected ALP, a related regulator with close connection of osteogenic differentiation. Our data showed that miR-9-5p bind the 3' UTR of ALP mRNA. Moreover, the dual-luciferase reporter assay showed that the luciferase activity of ALP 3' UTR was significantly decreased in miR-9-5p-transfected 293T cells. In addition, western blot also showed that the ALP level significantly decreased by miR-9-5p mimic and increased by miR-9-5p inhibitor in Figure 2, which is consistent with our suggestion. These data confirmed that ALP was a direct target of miR-9-5p (Figure 6).

## Discussion

The imbalance between osteoblasts and osteoclasts can induce osteoporosis 15. While hBMSCs can be induced into osteoblasts and improved ability of hBMSCs osteogenic differentiation are the key to bone regeneration 16. Therefore, it is necessary to develop alternative agents effectively to treat osteoporosis in older patients. Over the last 50 years, the omega-3 family had two superstars: EPA and DHA 17. EPA stands for eicosapentaenoic acid, and DHA is the acronym for docosahexaenoic acid. Naturally found in fish and breast milk, EPA and DHA are considered the most potent omega-3, and higher intakes of EPA and DHA might reduce the risk of coronary heart disease 18. Because of their profound influence at the cellular level, EPA and DHA are instrumental for the healthy growth, development, and maintenance of cells and tissues in our body 19.

Studies assure that DHA is supposed to potentiate osteogenic differentiation 12. DPA is lesser-known omega-3 family member and gains more attention as researchers start to uncover its benefits. Like EPA and DHA, DPA is also classified as a “very long-chain fatty acid” and is predominantly found in fatty fish, seal meat, and breast milk. It also has the same number of carbon atoms as DHA and the same number of double bonds as EPA 20. Like EPA and DHA, DPA is important for improving lipid metabolism, decreasing aortic plaque build-up, and reducing chronic inflammation 21. Nevertheless, the mechanism of action of DPA in hBMSCs remains ambiguous. Here, we determined whether DPA could play a crucial role in osteogenesis and its underlying mechanisms. Our research demonstrated that DPA promoted osteogenesis and regulated the osteoblastic differentiation by the miR-9-5p/ERK/ALP pathway.

In this study, we first focused on the *in vitro* effects of DPA on the differentiation of hBMSCs. *In vitro* experiments, the expression of ALP was significantly increased as well as highly expressed OPN and OCN by qPCR and Western blotting at 20 µM concentrations, which indicating the DPA could enhance osteoblast viability and promote osteoblast differentiation via ALP.

miRNAs play important regulatory roles in regulating the osteogenic differentiation of BMSCs 22. To investigate the mechanism of DPA impacts on osteogenesis, we conducted a miRNA sequence. Many differentially expressed miRNAs were identified after treated with DPA, including miR-9-5p, miR-33a-5p, and miR-197-3p. However, only the expression of miR-9-5p was significantly decreased was observed after 14 days of DPA induction. miR-9-5p was overexpressed in high glucose treated BMSCs and inhibited high glucose induced osteogenic differentiation BMSCs *in vitro* and *in vivo* 23. In our study, we first found that the overexpression of miR-9-5p could strongly inhibit osteogenesis of hBMSCs. In addition, to study the molecular mechanism by which miR-9-5p regulates the differentiation of hBMSCs, we found that ALP as a direct target of miR-9-5p via a dual luciferase reporter assay. ALP is a key factor for osteoblast activation during the early stages of osteogenic differentiation. Thus, our findings provide new insight into the regulation of osteogenesis of miR-9-5p.

Researchers have shown that phosphorylated ERK is intensively activated during osteogenic differentiation and can activate the expression of Runx2 24. We found that DPA significantly increased the ERK phosphorylation while miR-9-5p inhibited the activity of ERK in hBMSCs. While PD98059, a specific ERK inhibitor, and miR-9-5p antagonist abolished the DPA induced activation of ERK and inhibited the promotion effect of osteogenic differentiation. These data indicated that ERK pathway was involved in the biological effects of DPA/miR-9-5p signaling pathway.

## Conclusion

**Figure 7:**
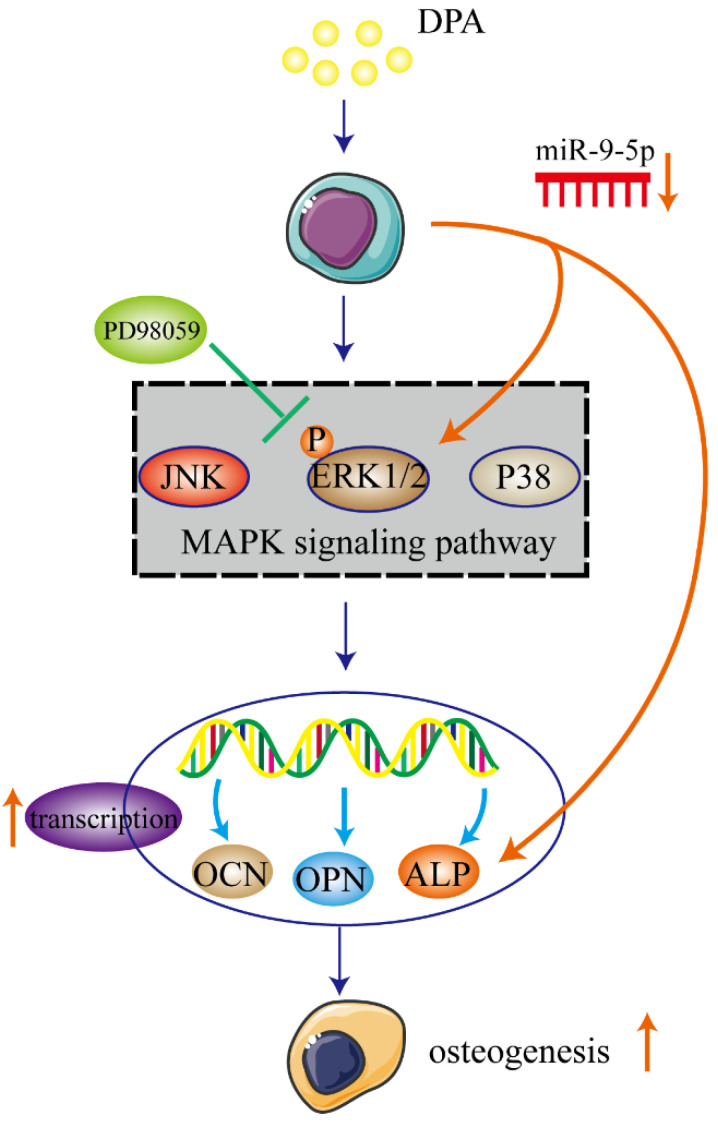
Schematic illustration of potential signaling pathways associated with the osteogenic effects of DPA.

In the present study, we demonstrated that DPA induced osteogenic promotion in hBMSCs with miR-9-5p and activating ERK signaling pathway via ALP. DPA might be a potential therapeutic agent for treatment of osteogenesis (Figure 7).
